# City Dweller Responses to Multiple Stressors Intruding into Their Homes: Noise, Light, Odour, and Vibration

**DOI:** 10.3390/ijerph120303246

**Published:** 2015-03-18

**Authors:** Eja Pedersen

**Affiliations:** Environmental Psychology, Department of Architecture and Built Environment, Lund University, P.O. Box 118, SE-221 00 Lund, Sweden; E-Mail: eja.pedersen@arkitektur.lth.se; Tel.: 46-46-222-32-41

**Keywords:** environmental stressors, noise, vibration, odour, light, annoyance, place attachment, general health, sensitivity, quality of life

## Abstract

Urban densification increases exposure to noise, light, odour, and vibration in urban dwellings. Exposure from combined environmental stressors intruding into the home could increase the risk of adverse effects on wellbeing, even when the exposure is at a relatively low level. This study assesses the prevalence of annoyance with a combination of potential environmental stressors common in urban areas and the association with wellbeing. A questionnaire was sent by mail to residents in five areas in Halmstad (Sweden) with similar socioeconomic and housing characteristics but different exposure (response rate 56%; *n* = 385). Of the respondents, 50% were annoyed to some degree by at least one of the suggested stressors, most commonly by noise and vibration from local traffic. Structural equation modelling showed that annoyance led to lowered quality of life via the mediating construct residential satisfaction, which in turn was influenced by place attachment and perceived restoration possibilities in the dwelling. Stress had a negative impact on quality of life, but was not directly correlated to annoyance. Stress was however correlated with sensitivity. The findings suggest that dose-response relationships for environmental stressors should be studied in a broader context of environmental and individual factors. Also relatively low levels of exposure should be mitigated, especially if several stressors are present.

## 1. Introduction

Most health impact assessments and guidelines originate from the idea that people are exposed to single pollutants [1]. In reality, people are often simultaneously subjected to a range of multiple environmental stressors, possibly resulting in attenuated, additive, or even potentiated effects [2]. Humans are equipped with multiple sensory channels through which we experience the environment [3] and have the capacity to combine sensory inputs across modalities [4]. Exposures occurring simultaneously and affecting more than one sensory system may trigger a crossmodal process in the brain [4], engendering greater impact than that caused by single pollutants. Experimental studies have demonstrated that acute physiological stress reactions, in the form of raised neuroendocrine and endocrine hormone levels, heart rate, and blood pressure, are stronger when people are exposed to more than one stressor [5,6]. The effects could also be viewed psychologically in light of Lazarus and Folkman’s [7] cognitive stress theory. When a person is confronted with a potential stressor, a process starts in which the stressor is appraised either as of no concern or as something disturbing, threatening, or even harmful. If the situation is not dismissed as of no concern, the individual explores his or her ability to cope with the new conditions in a secondary appraisal. Adaptive cost theories suggest that coping with a stressor can reduce the capacity to cope with subsequent stressors [2]. Despite their physiological or psychological basis, responses to multi-sensory exposures and their consequences for life quality of the residents are poorly understood [8].

Early studies of the effects of multiple stressors focused, with some exceptions, on *one physical stressor* combined with *stressful conditions* such as stressful life events [9] and job stress [10]. Studies of *one type of physical stressor*, such as noise, from *multiple sources* have produced ambiguous results with, for example, attenuated [11], additive [12], and even masking effects [13] being identified. The nature of effect can even differ between sound level intervals [14,15]. Studies of *two physical stressors from the same source* were reviewed by Lercher [1]. In the case of railway noise and vibration, there seem to be a moderating effect, though there is no overall clear trend. More evidence is available of the synergetic effects of combined exposure to noise and air pollution. Responses to *several physical stressors from different sources* have, to our knowledge, not been extensively studied. A large study in a general population found several sources of annoyance (e.g., traffic noise, street and nightlife noise, and wood burning odour) and for some of these a dose-response relationship, though without further links to well-being or quality of life [16]. There is, as pointed out [8], a need for additional studies concerning the effects of combinations of environmental stressors.

If synergistic effects exist, it means that exposures to relatively low levels of multiple environmental stressors could have a negative impact. This may explain why some studies have found that interference arises at levels not expected to yield any negative response. Wind turbines generate low levels of noise, compared with other noise sources, but nevertheless lead to annoyance in some situations, a fact partly explained by the simultaneous visual exposure to rotating blades [17]. In these cases it may be difficult to relate the response to some form of dose, as the influence of local factors will be larger than at higher exposure levels. In addition, it is difficult to find appropriate dose metrics for some environmental stressors such as light pollution. A starting point may instead be people’s experiences of environmental stressors that generate negative responses in form of reported annoyance that in turn have an adverse impact on quality of life.

Quality of life has been defined by WHO as “a person’s perception of his/her position in life within the context of the culture and value systems in which he/she lives and in relation to his/her goals, expectations, standards and concerns” [18], which has been understood as the extent to which individuals’ vital needs are satisfied [19]. The concept comprises several aspects such as health, safety and social relations [20] of which health has been most commonly used as the endpoint in exposure-response studies [21,22]. There seem to also be a more general idea of what quality of life is, described and measured as life satisfaction [23]. Life satisfaction could change over time due to external events, but is also related to individual resources and hence is rather stable [24]. Experiences of daily stressors such as work deadlines or family needs affect well-being more immediately and varies therefore over shorter time [25]. Studies concerning environmental stressors’ impact on quality of life would probably benefit from including measurements that capture a more general aspect of quality of life (life satisfaction) in addition to the health aspect, complemented by the current state of perceived stress.

An individual factor that is well known to influence the risk for noise annoyance is noise sensitivity [26], defined as strong reactions to specific noise situations [27] or as a personality trait [28]. A definition adopted by several researcher within the field, (e.g., [29]) was suggested by Job [30]; noise sensitivity refers to the internal states of an individual (physiological, psychological, or life-style determined), which increase their degree of reactivity to noise in general. Noise sensitivity has been found to increase annoyance independently of noise exposure [31]. There are indications that there are similar relationships between sensitivity and annoyance for other senses though they are not as commonly studied [32]. It has been debated if a general environmental sensitivity exists or not [32,33]. In any case, self-reported sensitivity to air pollution, noise, and odour were in a previous study found to correlate strongly but not totally [34].

The experience of environmental stressors intruding into the dwelling takes place in a specific physical environment, a home, which is not just an objective but also an emotional place [35]. The strength of an affective link between the person and his or her environment [36] has been thoroughly explored since the 1970’s but seldom in relation to exposure of physical stressors [37]. A core concept is place attachment, a positive bond that develops between people and their environment, which is not the same as residential satisfaction [38]. One may be satisfied with the dwelling and the neighbourhood without being particularly attached, and vice versa. Place attachment is known to be related to both physical and psychological quality of life [39]. It has been proposed that an individual become attached to a place because it has restorative qualities that support self-regulation (enabling positive emotional changes and renewal of cognitive capacities) [40]. In the light of the cognitive stress theory, individual’s relation to place and perception of how well the place meet restorative needs should hence be included in studies of environmental stressors intruding into the home.

This study addresses medium-level environmental external stressors intruding into urban dwellings. The objectives of the study were to assess the

(i) prevalence of annoyance with multiple stressors (in this case noise, light, odour, and vibration, from traffic and stationary sources) under medium-level exposure
(ii) prevalence of annoyance in residential areas with different patterns of low to medium exposures from single *vs.* multiple sources
(iii) relationship between studied exposures and quality of life in terms of life satisfaction and general health
(iv) contributions of self-reported sensitivity, stress, residential satisfaction and place relation in the causal chain leading from exposure to quality of life

It was predicted, based on research reviewed above, that quality of life would be influenced by annoyance (negatively), place relation (positively), residential satisfaction (positively), daily stress (negatively) and recovery needs (negatively). Sensitivity was expected to enhance annoyance.

## 2. Method

### 2.1. Study Areas and Samples

**Figure 1:**
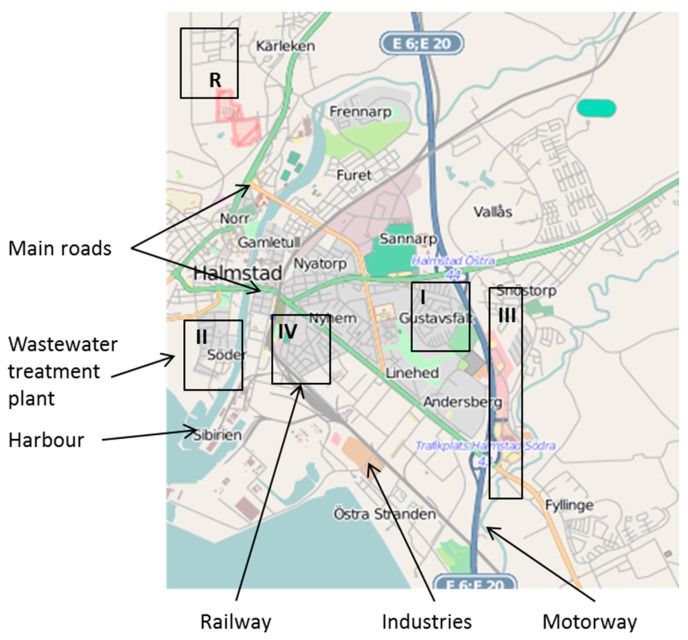
Map of Halmstad showing the five investigated areas and potential stressor sources.

The study was conducted in Halmstad, a city of 62,000 inhabitants located on Sweden’s southern coast. To capture a variety of responses, four neighbourhoods near potential sources of environmental stressors were selected from maps (areas I–IV). A reference area considered relatively free of exposure (area R) was also included for comparison. The five areas were located within 6 km of the city centre (*i.e.*, the main square) of Halmstad (Figure 1) and had the same urban character, comprising mainly detached, semi-detached, or terraced houses. In all areas, the majority of the residents had private gardens with direct access from the dwelling. The socioeconomic status of the areas could be considered middle class in a Swedish context, with residents likely having enough resources to move elsewhere if they preferred; a spatial selection based on perception of environmental stressors could have occurred before the study. However, a parallel study found no indications of such a selection [41].

The exposed areas differed in distance to various sources that could be expected to give rise to indoor stressor exposures, *i.e.*, industry, a sewage treatment plant, a harbour, roads, and railway tracks. The areas were visited at different times of day to determine the exposure situation. Local residential traffic at low intensities was present in all five areas. No other potential sources of annoyance were identified in the reference area (R). In areas I and III, noise from the motorway was present at all times of day and night, in area III enhanced by road traffic noise from a main road and a shopping mall parking lot. The noise situation was nevertheless not classified as problematic by the authorities, so the exposure could be regarded as of medium level. The traffic was sheltered in area I, but highly visible in area III in which vibrations from trucks also could be sensed. In area II, no smell from the sewage plant was identified during any of the visits, but the odour problem was confirmed by local authorities, which had received complaints. The same was true of area IV regarding the nearby industrial site. Area IV was also clearly dominated by its proximity to the train station (including a shunting yard) and bus station. Control measurements of A-weighted maximum sound pressure levels, *L*_Amax_, near dwellings in the area indicated levels of 68–101 dBA when freight trains passed at night. In summary, area I was exposed to one kind of exposure (auditory), area II to two kinds (auditory, olfactory), area III to three kinds (auditory, visual, vibratory), and area IV to four (auditory, vibratory, visual, olfactory) (Table 1).

**Table 1:** Characteristics of the study areas; all areas were exposed to low intensity local traffic.

Characteristics	R	I	II	III	IV
Overall exposure	Low	Medium	Medium	Medium	Medium
Sources	-	Single	Multiple	Multiple	Multiple
Sensory input	-	Auditory	Auditory and olfactory	Auditory, visual, and vibratory	Auditory, vibratory, visual, and olfactory
Main source	-	Motorway (one direction, not visible)	Sewage treatment plant Harbour	Motorway Main road Shopping mall	Railway tracks Shunting yard Bus depot Industry

Addresses of residents aged 18 years or older in the five selected areas were requested from the municipality. In some areas, there were apartment buildings whose residents were removed from the list if they could be identified based on their addresses. The selection of potential participants was then made in two steps: (i) one person per household was randomly selected; (ii) from these selected subjects, 100 in the reference area and 150 in each of the other areas were randomly selected, resulting in a study sample of 700 persons.

### 2.2. Measurements of Annoyance

A questionnaire was used to measure the perceived existence and experience of 15 specified environmental stressors, *i.e.*, air pollution, industrial odour, sewage odour, train vibration, bus and truck vibration, street lighting, road traffic headlights, train headlights, mast lights, exterior lighting or signs, passing car noise, road traffic noise, recycling station noise, moped or motorcycle noise, and train noise. Response to environmental stressors was prompted by the following instructions: “Indicate for each of the following inconveniences whether you notice or are annoyed by them while staying indoors in your home”, using a five-point verbal rating scale (VRS) in which 1 = “do not notice”, 2 = “notice but not annoyed”, 3 = “slightly annoyed”, 4 = “rather annoyed”, and 5 = “very annoyed”. This scale has previously been used in several community noise studies (see Pedersen [42]). An *annoyance index* was calculated for each respondent as the sum of all 15 ratings, hence scores could range from 15 to 75. The index reflects both the number of sources that were perceived as annoying and how severe they were perceived as being, though it does not distinguish between these two dimensions. The index was therefore complemented with two alternative variables, *i.e.*, the number of sources perceived as at least somewhat annoying (rating 3–5) [43], ranking from 0–15, and the number of senses (noise, light, odour, and vibration) related to the annoyance, ranking from 0–4.

### 2.3. Measurements of Quality of Life

Quality of life was captured by two dimensions. *General health* was measured using a single question, “How would you rate your general health?”, answered using a five-point VRS: 1 = “very good”, 2 = “good”, 3 = “neither good or bad”, 4 = “bad”, and 5 = “very bad” [22]. The scale was reversed during analysis so that a high score corresponded to good health. *Life satisfaction* was measured using five items, *i.e.*, (1) “On the whole, my life is exactly as I want”; (2) “My living conditions are excellent”; (3) “I am satisfied with my life”; (4) “Given my age, I have so far got the important things I want out of life”; and (5) “If I could live my life over, I would change almost nothing”, responded to on a seven-point VRS ranging from 1 = “strongly disagree” to 7 = “agree precisely”. These items have previously been used and validated [23,44].

### 2.4. Measurements of Possible Moderators or Mediators

The questionnaire also included measurements of factors assumed to be involved in the relationship between annoyance and quality of life. *Sensitivity* to noise, odour, and vibration was measured using one item for each of these three categories of stressors on a four-point VRS: 1 = “not sensitive”, 2 = “not particularly sensitive”, 3 = “quite sensitive”, and 4 = “very sensitive”; a sensitivity index was created by averaging the scores (Cronbach’s alpha = 0.816) [17,34]. Perceived *stress in daily life* was measured using a single question, *i.e.*, “How often do you feel under stress in your everyday life?”, the response options ranging from 1 = “never” to 5 = “very often”. This question was followed by a question intended to capture the *need for stress recovery*, *i.e.*, “How great a need to recuperate do you estimate that you have in your everyday life?”, answered using a VRS ranging from 1 = “none” to 5 = “very great”. These two questions on stress were developed for this study. Perceived *restoration possibility* was assessed using one item, *i.e.*, “I feel that I can take it easy and relax in my home”, with response options ranging from 1 = “strongly disagree” to 5 = “agree precisely” [45]. *Satisfaction with the dwelling* and *satisfaction with the neighbourhood* were each assessed using a single question, with response options ranging from 1 = “very low” to 5 = “very high” developed for this study. *Place attachment* was measured using five items, *i.e.*, (1) “I might as well live elsewhere”; (2) “I feel nothing special for this home”; (3) “I feel as if I belong in this house”; (4) “I feel emotionally attached to this residence”; and (5) “I identify with this home”, also responded to on a five-point VRS ranging from 1 = “strongly disagree” to 5 = “agree precisely”, reflecting different levels of attachment [35]. An index was created by inverting the responses to the first two items and calculating the average of all five items for each participant (Cronbach’s alpha = 0.868). Socio-demographic data (*i.e.*, age and gender) were also obtained using the questionnaire.

### 2.5. Handling of and Response to the Questionnaire

The questionnaire was sent by mail to 700 persons in February 2012 with a reminder sent in March 2012. Fourteen of the questionnaires were returned because the addressee had moved or was deceased. Of the 686 people who most likely received the questionnaire, 385 properly completed and returned the form (see Table 2). The response rate was 55% or higher in all areas except in Area IV, where it was 48%.

**Table 2:** Questionnaire administration and results.

Characteristics	R	I	II	III	IV	Total
Sent	100	150	150	150	150	700
Returned	4	3	5	1	1	14
Respondents	61	87	83	83	71	385
Response rate, %	64	59	57	56	48	56

### 2.6. Data Analyses

For ordinal and continuous data, the distribution of variables is presented using arithmetic means and standard deviations, while for nominal data it is presented using percentages. For ordinal and continuous data, differences between areas were tested using one-way analysis of variance (ANOVA) (two-sided), followed by the post-hoc least significant difference (LSD) test if differences were found. For nominal data, differences were tested with Kruskal-Wallis nonparametric test. Correlations were tested with Pearson’s moment correlation test (two-sided). For all tests, *p*-values below 0.05 were considered as indications of statistical significance.

Structural equation modelling was used to assess more complex relationships between the studied variables with a two-step approach [46]. In the first step, a measurement model was tested to ensure that the manifest variables loaded satisfactorily on their respective latent variables and to explore the strength of the relationships between variables. In the next step, possible causal relationships were tested, building on the pathway *Area* (as proxy for exposure), *Annoyance* and *Quality of life*, and the other variables as possible mediators or moderators. The model was based on the theoretical framework reviewed in the Introduction, and further developed based on correlation strengths in the measurement model, modification indices and model fit. Pathways or correlations that did not reach statistical significance were deleted underway. Three indices of how well the model fitted the data were used:

(i) normed χ^2^, which is the ratio between χ^2^ (magnitude of discrepancy between the sample covariance matrix and the estimated covariance matric) and its degrees of freedom with a value <3.00 considered as an acceptable fit [47]
(ii) comparative fit index CFI which indicates how well the covariance of the data is captured; a value >0.90 is acceptable [48], and
(iii) root mean square error of approximation RMSEA which is the discrepancy per degree of freedom for the model; values <0.06 indicates close fit to the data [49]

This approach could result in a somewhat better fit than if the modelling is purely driven by theory, but was in this study motivated by the attempt to use concepts from several disciplines not usually combined. The statistical tests were carried out with the software IBM SPSS Statistics 22 and Amos 22.

## 3. Results

### 3.1. Annoyance Related to Area

The mean annoyance score for the total response group was 21.8, ranking from 18.0 to 26.5 in the five areas (Table 3). Fifteen percent were annoyed (somewhat or more) by at least one of the 15 potential stressors in their homes. The highest frequencies of annoyance were found for vibration from buses or trucks (23%), noise from passing cars (22%), noise from mopeds and motorbikes (20%), and motorway noise (17%). The distribution of annoyance with specified sources between areas was consistent with observations. Annoyance due to local road traffic was present in all areas, while annoyance due to distant road traffic, trains, or industry was found only near these sources. The highest percentages of annoyance due to distant traffic were found in areas I (52%) and III (26%); this was expected, as both areas are situated near the motorway. Area III also reported annoyance due to vibration from buses and trucks (32%). Vibration was likewise a problem in area IV (34%) near the bus depot. The highest percentages of annoyance due to air pollution (31%) and odour from industry (49%) were found in area IV near an industrial site. Odour from wastewater treatment was reported as annoying in area II (30%) near the sewage plant. Annoyance with train noise (45%) and vibration (46%) was found almost exclusively in area IV. As expected, respondents living in multi-exposed areas (*i.e.*, II, III, and IV) were more often annoyed by more than one stressor source than were those living in the reference, area R, or area I exposed mainly to road traffic noise (Table 3).

No relationship between the annoyance score and age (*r* = −0.085, *p* = 0.097), or between annoyance score and gender (*F*(1,380) = 3.24, *p* = 0.073), was found.

**Table 3:** Annoyance due to environmental stressors in the home in the five study areas and in total.

Characteristics	R	I	II	III	IV	Total
	*n* = 61	*n* = 87	*n* = 83	*n* = 83	*n* = 71	*n* = 385
Annoyance score **^a^**	18.0	19.3	22.0	23.1	26.5	21.8
*Annoyed by at least*						
One source, %	18	37	51	63	78	50
Two sources, %	10	21	41	46	56	35
Three sources, %	8	9	27	33	44	24
Four sources, %	2	6	19	22	35	17

**^a^** *F*(4,380) = 18.55, *p* < 0.001. *Post-hoc* test LSD showed that all areas differed statistically significantly from each other except area R and area I.

### 3.2. Annoyance Related to Stimulated Senses

Of the 15 potential stressors, five concerned noise, two vibration, three odour or air pollution, and five light exposure. Of all respondents, 37% were annoyed by one or more modes of environmental noise, 24% by vibration, 20% by odour, and 14% by light. This annoyance occurred in different combinations among the respondents annoyed by stimulation of two (13%), three (9%), or all four senses (4%) tested here.

**Figure 2:**
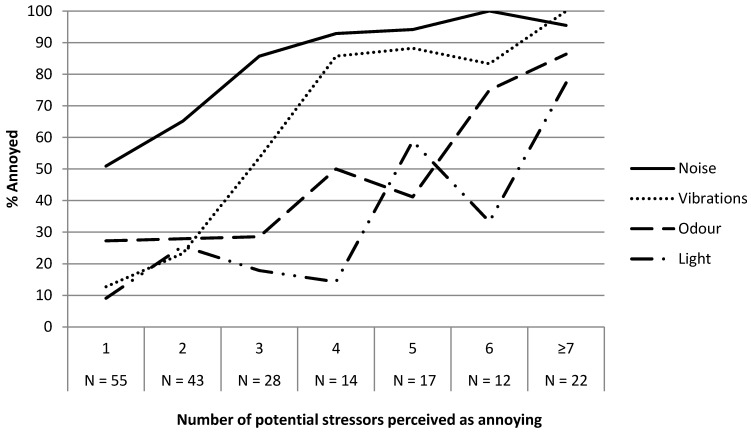
Proportions of respondents annoyed by noise, vibration, odour, and light relative to how many potential stressors they perceived as annoying.

Respondents annoyed by only one stressor (*n* = 55) were most often annoyed by noise (51%) or odour (27%) (Figure 2). Being annoyed by two or three stressors mainly increased the likelihood of perceiving *noise* and *vibration* as annoying. Among those annoyed by four or more potential stressors, more than half were annoyed by stressors stimulating at least three different senses.

### 3.3. Quality of Life and Potential Moderating and Mediating Factors Related to Area

Most respondents reported good general health and moderate or good life satisfaction (Table 4). As expected, some respondents reported being rather or very sensitive to various environmental exposures: 32% were sensitive to noise, 43% to odour, and 32% to vibration. The occurrence of stress in daily life and the need for stress recovery were intermediate. The dwellings were on average perceived as suitable for rest and relaxation. Satisfaction with the dwellings and neighbourhood as well as place attachment was high.

The results of reported annoyance on an area level was not reflected in measurements of quality of live or in related variables; the results were approximately equal in all areas, with some exceptions. General health was higher in areas R and II and lower in area III. Satisfaction with the neighbourhood was higher in area R and II than in the other areas. Respondents in area I were older than in the other areas.

**Table 4:** Variables measuring quality of life, sensitivity, stress, residential satisfaction, and place relation: mean values (with standard deviations in parentheses) for each area and in total.

Variables	R	I	II	III	IV	Total	Test of Differences, *p*-Values
	*n* = 61	*n* = 87	*n* = 83	*n* = 83	*n* = 71	*n* = 385	
*Quality of life*							
General health (1–5)	4.3 (0.72)	4.1 (0.83)	4.3 (0.74)	4.0 (0.89)	4.1 (0.75)	4.2 (0.75)	0.036 **^a^**
Life satisfaction (1–7)	5.4 (0.95)	5.2 (1.21)	5.2 (1.09)	5.1 (1.36)	5.1 (1.16)	5.2 (1.17)	0.636
*Sensitivity*							
Noise (1–4)	2.2 (0.99)	2.1 (0.82)	2.3 (0.84)	2.3 (0.74)	2.2 (0.79)	2.2 (0.83)	0.592
Odour (1–4)	2.3 (0.98)	2.1 (0.82)	2.5 (0.87)	2.4 (0.87)	2.3 (0.87)	2.4 (0.90)	0.650
Vibration (1–4)	2.0 (0.86)	2.1 (0.82)	2.2 (0.80)	2.4 (0.87)	2.3 (0.87)	2.2 (0.83)	0.670
*Stress*							
Stress in daily life (1–5)	2.8 (1.01)	2.5 (1.04)	2.8 (1.01)	2.8 (1.06)	2.9 (1.22)	2.7 (1.07)	0.345
Need for stress recovery (1–5)	3.1 (1.16)	2.8 (1.24)	3.3 (1.15)	3.0 (1.14)	3.2 (1.17)	3.1 (1.18)	0.111
*Residential satisfaction*							
Satisfaction with the dwelling (1–5)	4.8 (0.42)	4.7 (0.60)	4.6 (0.68)	4.6 (0.63)	4.6 (0.60)	4.7 (0.60)	0.313
Satisfaction with the neighbourhood (1–5)	4.8 (0.42)	4.4 (0.78)	4.7 (0.60)	4.4 (0.80)	4.3 (0.77)	4.5 (0.72)	0.001 **^b^**
*Place relation*							
Place attachment (1–5)	4.2 (0.78)	3.9 (0.62)	4.0 (0.88)	4.0 (0.89)	3.9 (0.82)	4.0 (0.85)	0.311
Restoration possibilities (1–5)	4.5 (0.70)	4.3 (0.73)	4.4 (0.78)	4.2 (0.87)	4.3 (0.89)	4.3 (0.80)	0.276
Age	53 (14)	57 (14)	51 (15)	52 (15)	49 (14)	53 (15)	0.009 **^c^**
Gender, % female/male	49/50	57/43	51/49	54/46	47/53	52/48	0.712

**^a^** Area II had statistically significant higher values than did areas I, III and IV, respectively. Area III had statistically significant lower values than did areas R and II, respectively; **^b^** Areas R and II had statistically significant higher values than did areas I, III and IV, respectively; **^c^** Area I had statistically significant higher values than did areas II, III, and IV, respectively.

### 3.4. Structural Equation Model

Initial analyses showed that general health and life satisfaction were more correlated to each other than to any of the other factors (Table 5). The three items measuring sensitivity were strongly correlated, as well as the two items measuring stress and the two items measuring residential satisfaction. Perceived possibility to relax in the dwelling was more correlated to place attachment than to any of the other variables. However, place attachment was also correlated to the items measuring residential satisfaction.

**Table 5:** Correlations between variables measuring quality of life, sensitivity, stress, residential satisfaction and place relation.

Variables	1	2	3	4	5	6	7	8	9	10
1. General health										
2. Life satisfaction	0.402 **									
3. Sensitivity to noise	−0.004	−0.070								
4. Sensitivity to odour	−0.071	−0.071	0.622 **							
5. Sensitivity to vibration	−0.037	−0.076	0.688 **	0.702 **						
6. Stress in daily life	−0.101 *	−0.239 **	0.099	0.120 *	0.068					
7. Need for stress recovery	−0.084	−0.217 **	0.108 *	0.113 *	0.075	0.601 **				
8. Satisfaction with the dwelling	0.228 **	0.378 **	−0.024	−0.021	−0.048	−0.151 **	−0.104 *			
9. Satisfaction with the neighbourhood	0.231 **	0.347 **	−0.167 **	−0.085	−0.124 *	−0.071	−0.066	0.598 **		
10. Place attachment	0.084	0.317 **	0.005	0.033	0.006	−0.114 *	−0.026	0.565 **	0.502 **	
11. Restoration possibilities	0.193 **	0.344 **	−0.032	−0.005	−0.046	−0.160 **	−0.076	0.411 **	0.431 **	0.550 **

** Correlation is significant at the 0.01 level; * Correlation is significant at the 0.05 level.

A measurement model, based on the theoretical framework and the correlation table, was set up, comprising five latent variables: *Quality of life* (general health and life satisfaction), *Sensitivity* (to noise, oudour and vibration), *Stress* (stress in daily life and need for stress recovery), *Residential satisfaction* (dwelling and neighbourhood), and *Place relation* (place attachment and restoration possibilities). Also included was area, as proxy for exposure, and the annoyance score. All five latent variables as well as area and annoyance were initially allowed to correlate. The test of the measurement model resulted in good fit indices (normed χ^2^ = 1.57; CFI = 0.98; RMSEA = 0.04 (90% CI: 0.02 to 0.06)) and showed that all manifest variables (*i.e.*, variables measured by the questionnaire) loaded satisfactorily on their respective latent variables (standardized regression weights from 0.67 to 0.86), except for general health that loaded somewhat low on quality of life (0.47).

**Figure 3:**
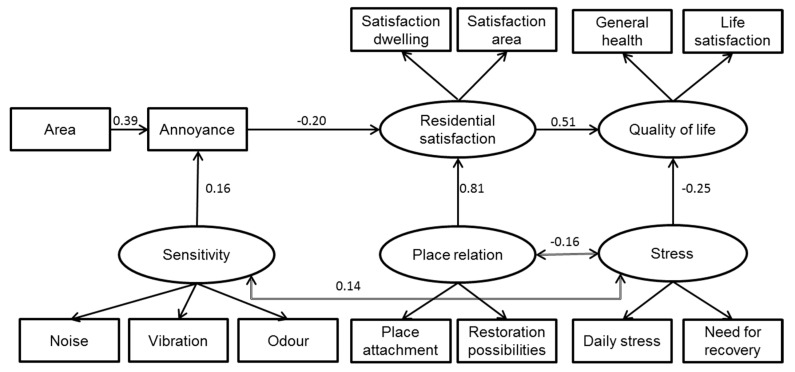
Structural equation model of the relationship between area, annoyance and quality of life, with mediating and moderating variables.

In the final structural equation model, area predicted annoyance that in turn had a negative impact on residential satisfaction (Figure 3). Residential satisfaction acted as a mediator to quality of life, so that high residential satisfaction increased quality of life. No direct effect of area or annoyance on quality of life was found. Annoyance was influenced by sensitivity, but not by any of the other studied variables. Sensitivity was correlated to some degree with stress. Stress had a direct negative impact on quality of life and was also correlated to place relation. Place relation was a strong predictor of residential satisfaction. Neither place relation nor residential satisfaction was related to area. Standardized direct effects of residential satisfaction and stress on quality of life correspond to regression weights in Figure 3. Indirect effects on quality of life were for area 0.03, annoyance 0.08, sensitivity 0.01, and place relation 0.42. The good fit between model and data from the test of the measurement model was kept (normed χ^2^ = 1.71; CFI = 0.97; RMSEA = 0.04 (90% CI: 0.02 to 0.06)).

In the model, 36% of the variance in quality of life was explained. The main part of this could be traced back to place relation (standardized indirect effect 0.41), but also annoyance contributed (standardized indirect effect −0.10), via the mediator residential satisfaction. In turn, 18% of the variance in annoyance score was explained by which area the respondent lived in (standardized direct effect 0.39) and self-estimated sensitivity (standardized direct effect 0.16).

The model was finally tested with the two alternative annoyance variables instead of the annoyance index: number or sources perceived as annoying and number of types of sources. The standardized regression weights were almost identical with that described for the annoyance score for number of sources (area to annoyance 0.39, annoyance to residential satisfaction −0.21, residential satisfaction to quality of life 0.51) and number of types of sources (area to annoyance 0.38, annoyance to residential satisfaction −0.18, residential satisfaction to quality of life 0.51) The model fit indices were also similar for the two alternative annoyance scales number of sources (normed χ^2^ = 1.67; CFI = 0.97; RMSEA = 0.04 (90% CI: 0.02 to 0.06)) and number of types of sources (normed χ^2^ = 1.65; CFI = 0.98; RMSEA = 0.04 (90% CI: 0.03 to 0.06)) as for the annoyance score.

## 4. Discussion

In the studied, rather ordinary, urban areas, half of the respondents reported being at least somewhat annoyed by one or more stressors intruding into their homes, and more than a third were annoyed by at least two sources. Most respondents were not severely annoyed, but the results are nevertheless alarming considering that the exposure levels were well below those prompting action by authorities. Noise was the most common type of stressor reported in line with other population studies [50]. However, the more sources of environmental stressor in a residential area, and thus more sources that are considered annoying, the greater the risk that more than one sensory mode becomes affected. The model that explored the relationship between annoyance and quality of life was unaffected by which measure of annoyance that was used, suggesting that the number of stressors perceived as annoying, or the type of stressors in relation to sensory mode, is as important as the severity of annoyance. The results support the initiatives in noise research advocating that other environmental stressors are studied in the same way as noise [51], and that the combined effect is taken into account.

One difficulty is to find adequate exposure measures and that was also the major problem in this study. Established methods that calculate the sound level at the façade of a residential on the basis of traffic intensity is not suitable at low levels where small differences in the design of the building as well as the resident’s life style can determine how much noise actually reaches the individual. Measurements in each dwelling, preferably long term, would give a better, but more costly, understanding of the dose. Doses of air pollution could also be assessed by modelling even though there is no consensus on which emission to capture [16,51]. Attempts to link odour to annoyance show that the individual differences in how pleasant different odours are judged impact the dose-response relationship [52]. There is thus a need to find new ways to capture exposure to study the effects of multiple exposures. This article demonstrates a possibility which, however, needs further study.

The demonstrated link between area and annoyance failed to further link annoyance to quality of life in the analyses of the sub-samples in the five areas. More subtle analyses using structural equation modelling showed that there was a connection between exposure and quality of life, mediated by annoyance. The results were in line with previous studies of noise impact that have commonly found that self-reported health or wellbeing is more strongly related to noise annoyance than directly to exposure levels [53,54]. Also expected was the impact of sensitivity on annoyance, at least when it comes to noise. It is well established that possibly about a third of the population in the Western world is more sensitive to noise than are others, and that this sensitivity is partly inherited [55]. The prevalence of sensitivity to other environmental stressors in a population is not as well explored. Sensitivity to odour has mainly been studied as hypersensitivity (corresponding to hyperacousis), and its reported prevalence is therefore lower than that of noise sensitivity. However, a cross-sectional study, in which sensitivity was measured using the short version of the Chemical Sensitivity Scale, classified 33% of the population as generally odour intolerant [56], comparable to the 43% who reported odour sensitivity in the present study. Sensitivity was in the model included as an ordinal scale variable, and not dichotomised into sensitive and non-sensitive respondents, acknowledging that sensitivity is a gliding scale and concerns a large part of the population to some degree. Tests of the proposed model in subsamples representing sensitive versus non-sensitive respondents showed no influence of sensitivity (data available on request).

The relationship between annoyance and quality of life was in the structural equation model in the present study mediated by residential satisfaction, in turn largely influenced by place attachment. Expressed in terms of the appraisal process, stressors are dismissed as of no concern or treated as threatening well-being on basis of not just the stressor, but the total environment. Also other factors are of importance. Küller’s Human-Environment-Interaction model states that the outcome of such an emotional process in a given situation is governed by the individual’s perception of the physical environment and the social climate, in light of the person’s individual resources and of the activity that takes place [57]. A more comprehensive understanding of adverse effects of environmental stressors should hence take into account all these four dimensions. Activities such as resting in the home could be an especially vulnerable situation, reflected in the impact of perceived restoration opportunities on place relation in the present study. Individual resources were in this study represented both as sensitivity and stress state. The social climate was poorly captured (even though it could to some extent be included in residential satisfaction) and should be measured in future studies.

A cross-sectional study like this cannot establish a cause-effect relationship, and from a researcher’s perspective, future longitudinal studies are needed, possibly focusing on the effects when several senses are stimulated. From a societal perspective, the results from the present study nonetheless encourage action, as it is clear that a rather large group in society is affected by environmental stressors entering their dwellings. Dwellings are private spaces in which all residents should have the right to relax and restore themselves without unwanted external intrusions. It is essential to mitigate environmental stressors, not just at high levels but also at medium levels, to meet the needs of all urban dwellers.

## 5. Conclusions

A non-negligible part of the population is disturbed by environmental stressors which intrude into their homes. Annoyance occurs even when the exposure levels do not exceed recommended limits, especially if multiple sources interact and more than one sensory system is stimulated. Integrating approaches from environmental medicine with theories of environmental psychology increased the understanding of how environmental stressors affect people's quality of life in this study. The research field would benefit from further integration of these two disciplines. An increased understanding of human responses to environmental stressors will hopefully lead to public initiatives that in turn lead to reduced loads on exposed individuals.
